# 冷诱导液液萃取-分散固相萃取净化-超高效液相色谱-串联质谱法同时测定鸡蛋中白僵菌素和4种恩镰孢菌素残留

**DOI:** 10.3724/SP.J.1123.2021.02015

**Published:** 2021-12-08

**Authors:** Bolin LIU, Man NI, Xiaomei SHAN, Ji’an XIE, Yanyu DAI, Cheng ZHANG

**Affiliations:** 1.安徽省疾病预防控制中心, 安徽 合肥 230601; 1. Anhui Provincial Center for Disease Control and Prevention, Hefei 230601, China; 2.安徽医科大学, 安徽 合肥 230032; 2. Anhui Medical University, Hefei 230032, China

**Keywords:** 分散固相萃取, 超高效液相色谱-串联质谱, 冷诱导液-液萃取技术, 白僵菌素, 恩镰孢菌素, 鸡蛋, dispersive solid phase extraction (DSPE), ultra-performance liquid chromatography-tandem mass spectrometry (UPLC-MS/MS), cold-induced liquid-liquid extraction (CI-LLE), beauvericin, enniatins, egg

## Abstract

新型生物毒素白僵菌素(BEA)和恩镰孢菌素(ENNs)是由镰刀菌种产生的有毒代谢产物,主要污染谷物及其制品,会威胁人类健康,因此受到人们越来越多的关注。该工作建立了冷诱导液液萃取-分散固相萃取净化-超高效液相色谱-串联质谱法(CI-LLE-DSPE-UPLC-MS/MS)同时测定鸡蛋中白僵菌素和4种恩镰孢菌素残留的分析方法。以乙腈-水-乙酸(79:20:1, v/v/v)为提取溶剂,采用冷诱导液液萃取与分散固相萃取净化相结合的方法进行样品处理,同时,对影响待测物提取与净化效率的提取溶剂、冷冻萃取温度与时间、净化剂用量等因素和色谱条件进行了优化。样品经20 mL提取液涡旋提取20 min,放入-40 ℃冰箱静置30 min后,取2 mL上层溶液经70 mg C18粉末净化,离心,上清液于40 ℃浓缩至近干,残留物用1 mL 80%(v/v)乙腈水溶液溶解,进样分析。以乙腈与5 mmol/L甲酸铵溶液作为流动相进行梯度洗脱,经ACQUITY UPLC BEH C18色谱柱(100 mm×2.1 mm, 1.7 μm)分离,采用ESI^+^电离,在多反应监测模式下采集,白僵菌素采用稳定同位素内标法定量,4种恩镰孢菌素采用基质匹配曲线外标法定量。结果表明,5种待测物在0.1~50.0 μg/L范围内线性关系良好,相关系数(*r*^2^)为0.9983~0.9997,该方法的检出限(LOD)为0.05~0.15 μg/kg,定量限(LOQ)为0.20~0.50 μg/kg。以阴性鸡蛋样品为基质,在低、中、高3个浓度水平(0.5、5.0、25.0 μg/kg)下进行加标试验考察方法的准确度与精密度,各待测物的平均回收率为81.1%~106%,相对标准偏差(RSD)为0.27%~9.79%。采用所建立的方法对农村散养鸡蛋与市售鸡蛋进行检测,结果表明,BEA在散养鸡蛋的检出率为30.4%, 4种ENNs均未被检出。该方法灵敏度高,稳定性好,回收率高,定量准确,简单易操作,适用于禽蛋食品中白僵菌素与恩镰孢菌素的同时快速测定。

## Abstract (en)

Enniatins (ENNs) and beauvericin (BEA), known as emerging mycotoxins, are the toxic secondary metabolites produced by various *Fusarium* species. Most grain and grain-based products are contaminated with ENNs and BEA. Animals have been exposed to ENNs and BEA primarily due to consumption of cereal grains and cereal by-products. ENNs and BEA have been detected in animal-derived food and human breast milk, and they pose significant threats to public health. Therefore, more contamination data are urgently needed for the risk assessment of ENNs and BEA present in animal-derived food. To ensure the quality of animal-derived food, a method has been developed for the simultaneous detection of five emerging mycotoxins (viz. enniatin B, enniatin B1, enniatin A, enniatin A1, and beauvericin) in eggs by ultra-performance liquid chromatography-tandem mass spectrometry (UPLC-MS/MS) coupled with cold-induced liquid-liquid extraction (CI-LLE) and dispersive solid phase extraction (DSPE). The main factors governing the response, recovery, and sensitivity of the method, such as the type of extraction solvent, the temperature and duration of cold treatment in CI-LLE, the type and dosages of adsorbents, and apparatus conditions and the type of mobile phase used, were optimized during sample pretreatment and instrument analysis. The mycotoxin residues in eggs were extracted using 20 mL acetonitrile-water-acetic acid (79:20:1, v/v/v) mixture for 20 min by the vortex shock method. After mixing, the mixture was frozen for 30 min in a freezer at -40 ℃ and centrifuged for 10 min at 10000 r/min. A 2 mL aliquot of the upper acetonitrile layer was purified by using 70 mg of C18 adsorbents. After whirling, the mixtures were centrifuged at 10000 r/min for 5 min. The purified solution was then concentrated to nearly dry in nitrogen atmosphere at 40 ℃. The residues were dissolved in 1.0 mL 80%(v/v) acetonitrile aqueous solution. The target analytes were separated on an ACQUITY UPLC BEH C18 chromatographic column (100 mm×2.1 mm, 1.7 μm) at a column temperature of 40 ℃, with a flow rate of 0.3 mL/min. The injection volume was 5 μL, and gradient elution was conducted using acetonitrile and 5 mmol/L ammonium formate solution as the mobile phases. Multiple reactions monitoring (MRM) was conducted in the positive electrospray ionization (ESI ^+^) mode. The isotope internal standard method was used for quantification of BEA, and the matrix-matched external standard method was used for quantification of four ENNs. The results of the optimized method showed that the five analytes were completely separated by using the above-mentioned chromatographic column. Good linear relationships were obtained for the five mycotoxins in the concentration range of 0.1-50.0 μg/L; the correlation coefficient (*r*^2^) ranged from 0.9983 to 0.9997. The limits of detection (LODs) ranged from 0.05 to 0.15 μg/kg, while the limits of quantification (LOQs) ranged from 0.20 to 0.50 μg/kg. Accuracy and precision experiments were conducted by spiking egg samples with known amounts of analytes at three concentration levels (0.5, 5.0, and 25.0 μg/kg, in compliance with the current legislation) with six replicates. The average recoveries of the five analytes ranged from 81.1% to 106%, and the relative standard deviations (RSDs) were between 0.27% and 9.79%. The matrix effects of the analytes were between 2.70% and 45.1% in egg samples after pretreatment by CI-LLE coupled with DSPE. The developed method was applied to the determination of five mycotoxins in rural eggs and commercial eggs. BEA was detected in most rural egg samples, with detection rates of 30.4%. None of the four ENN residues were detected. Therefore, we can conclude that the method described herein has the advantages of sensitivity, stabilization, accuracy, good recovery, and easy operation, and is suitable for the simultaneous and rapid determination of BEA and ENN residues in eggs.

恩镰孢菌素(ENNs)和白僵菌素(BEA)是由镰刀菌属菌种产生的代谢产物^[1,2,3]^,主要污染谷物及其制品^[4,5,6,7]^。常见的恩镰孢菌素有恩镰孢菌素A(ENNA)、恩镰孢菌素A1(ENNA1)、恩镰孢菌素B(ENNB)和恩镰孢菌素B1(ENNB1)。近年来,随着研究的深入,动物性食品和人类母乳中均检出这两类毒素^[8,9,10,11]^。国外学者研究表明BEA和ENNs具有遗传毒性和细胞毒性^[12,13,14]^,有些毒性甚至超过先前发现的其他生物毒素,可能与其他生物毒素产生协同毒害作用^[15]^。目前,对于BEA和ENNs毒性的研究,国内少有报道^[16]^,且国标GB 2761-2017中还没有规定这两类毒素的限值。调查发现,我国玉米、小麦及其制品受到BEA与ENNs不同程度的污染,山东省部分地区玉米及其制品中BEA的检出率为82.3%, 4种ENNs的检出率为3.8%~56.3%^[17]^,韩小敏等^[18]^在从北京、安徽、宁夏等省(市/自治区)采集的市售玉米及其制品中同时检出BEA、ENNA、ENNA1与ENNB1,小麦及其制品中检出BEA和4种ENNs,其中ENNB与ENNB1的含量达到111.95和71.66 μg/kg,且发现小麦及其制品中的污染水平甚至高于玉米及其制品。玉米、小麦及其制品是动物养殖的主要饲料,动物食用高毒素污染的饲料后,增加毒素在动物组织或副产品中残留的风险,如牛奶与鸡蛋^[19]^。新兴生物毒素的污染引发的食品安全问题值得我们广泛关注,开展动物源性食品中BEA和ENNs污染水平的检测,为风险评估及制定BEA与ENNs的限值提供数据支持,同时,我省农村散养环节的鸡蛋中BEA和ENNs缺少例行监测,为此开展检测具有重要意义。

近年来,液相色谱-串联质谱法(LC-MS/MS)具有选择性好、抗干扰能力强、灵敏度高、二级质谱可有效排除假阳性、分析通量高等优势,成为多种毒素检测的首选方法^[20]^。目前,BEA和ENNs的检测方法有高效液相色谱法(HPLC)^[4]^和LC-MS/MS^[3,6-11,21]^,其中LC-MS/MS是主流分析方法,这些分析方法大多是以谷物及其制品、中草药与食用油为基质样品,结合液液萃取法^[9]^,固相萃取法^[8,18,22]^及QuEChERS法^[23,24]^等前处理技术建立的,针对动物源性食品中BEA和ENNs的检测方法^[8-9,22]^报道较少。动物源性食品的基质复杂,采用液液萃取法,提取液中的脂类等杂质较难除去,容易污染仪器,影响色谱柱寿命,而固相萃取法虽能够除去干扰杂质,但前处理步骤复杂,操作时间长,检测效率低,不适合批量分析。分散固相萃取(dispersive solid phase extraction, DSPE)法是结合液液萃取和固相萃取而建立的前处理技术,采用吸附剂吸附杂质,具有溶剂用量少、环保、经济、简单快速、灵敏度高等优点^[25]^。冷诱导液-液萃取技术(CI-LLE)是基于水与乙腈混合液在温度低于零度的条件下会发生相分离,极性强的化合物溶解在水相中,极性弱的化合物溶解在乙腈中^[26]^,而实现液液萃取的目的。该技术于1999年被Yoshida等^[27]^首次报道后,被学者不断研究和探索,与气相色谱-质谱法(GC-MS)、LC-MS/MS等检测技术联用测定食品中的有害物质^[28,29]^,应用领域进一步扩大。采用CI-LLE技术萃取后,将上层溶液再经DSPE方法净化,能显著降低基质效应的影响^[30]^。考虑到选取的5种待测物均属于低极性化合物,本实验采用CI-LLE与DSPE相结合处理样品,优化冷冻温度与时间、乙腈与水的比例及DSPE净化剂用量等前处理条件,提高了BEA与ENNs的提取与净化效率,充分发挥改良的DSPE法的优势,建立了超高效液相色谱-串联质谱法(UPLC-MS/MS)准确测定鸡蛋中BEA和4种ENNs毒素含量的分析方法。该方法灵敏度与准确度高、样品前处理步骤简便,经济实用,适用于禽蛋食品中BEA与ENNs的高通量快速分析。

## 1 实验部分

### 1.1 仪器与试剂

ACQUITY^TM^ UPLC超高效液相色谱-Xevo TQ质谱仪(美国Waters公司); Heidolph多点振荡器(德国Heidolph公司); Milli-Q超纯水制备仪(美国Millipore公司); Haier DW-86L33超低温冰箱(海尔生物医疗有限公司); Legend Mach 1.6R高速冷冻离心机(美国Thermo Fisher公司), 15 mL与50 mL聚丙烯塑料离心管(美国Thermo Fisher公司)。

甲醇、乙腈、甲酸与乙酸(均为色谱纯,德国Merck公司);乙酸铵、甲酸铵(色谱纯,美国Sigma-Aldrich公司); *N*-丙基乙二胺(PSA)与C18净化剂(均为50 μm,美国Agilent公司);氯化钠(分析纯,国药集团化学试剂有限公司);恩镰孢菌素A、恩镰孢菌素A1、恩镰孢菌素B与恩镰孢菌素B1纯度均大于99.0%,购自澳大利亚Bioaustralis公司;白僵菌素纯度大于99.0%,购自加拿大TRC公司;^13^C_45_-白僵菌素标准储备液购自青岛Pribolab公司,质量浓度为25 mg/L。

鸡蛋样品采集于本省不同地区的农村散养户、超市与农贸市场。

### 1.2 标准溶液的配制

分别称取恩镰孢菌素A、恩镰孢菌素A1、恩镰孢菌素B、恩镰孢菌素B1和白僵菌素1 mg(准确至0.01 mg),分别用乙腈溶解并定容至10 mL,配制100 mg/L标准储备溶液,-20 ℃下密封保存。

分别准确吸取5种标准贮备液100 μL于10 mL容量瓶中,加乙腈稀释至刻度,得到1 mg/L的混合标准使用液;吸取同位素内标(^13^C_45_-BEA)储备液400 μL于10 mL容量瓶中,加乙腈稀释至刻度,得到1 mg/L的同位素内标使用液,密封后于-20 ℃保存。

### 1.3 样品前处理

取10枚鸡蛋,去壳,混合均匀,准确称取2 g(精确到0.01g)混匀的蛋液于50 mL离心管中,加入25 μL同位素内标使用液,静置30 min后,加入20 mL乙腈-水-乙酸(79∶20∶1, v/v/v)提取液,涡旋振荡提取20 min,放入-40 ℃冷冻冰箱中静置30 min,然后在4 ℃下以10000 r/min离心10 min。移取2 mL上清液置于装有70 mg C18净化剂的15 mL离心管中,涡旋1 min后,10000 r/min离心5 min,收集净化液于另一个离心管中,40 ℃下氮气吹干,用1 mL乙腈水溶液(80∶20, v/v)溶解残留物,涡旋振荡1 min, 4 ℃下10000 r/min离心5 min,取上清液供UPLC-MS/MS分析。

### 1.4 分析条件

1.4.1 色谱条件

色谱柱:Waters ACQUITY UPLC BEH C18柱(100 mm×2.1 mm, 1.7 μm),保护柱(5 mm×2.1 mm, 1.7 μm);柱温:40 ℃;样品室温度:10 ℃;流速:0.3 mL/min;流动相A: 5 mmol/L甲酸铵水溶液;流动相B:乙腈;梯度洗脱程序:0~3.0 min, 35%A~5%A; 3.0~4.0 min, 5%A; 4.0~4.5 min, 5%A~35%A; 4.5~6.0 min, 35%A。进样量:5 μL。

1.4.2 质谱条件

离子源:电喷雾电离(ESI^+^)源;毛细管电压:3.0 kV;离子源温度:150 ℃;脱溶剂气温度:500 ℃;脱溶剂气流量:800 L/h;碰撞气流量:0.12 mL/min;多反应监测(MRM)模式。待测物的母离子、子离子及对应的碰撞能量、锥孔电压等质谱参数见表1。

**表1:** 5种待测物及白僵菌素同位素内标的质谱参数

Analyte	Abbreviation	Mode	t_R_/ min	Parent ion (m/z)	Product ions (m/z)		Collision energies/eV	Cone voltage/V
Enniatin B (恩镰孢菌素B)	ENNB	[M+NH_4_]^+^	2.72	657.3	86.1/196.2^*^	/214.2	60/30/30	40
Beauvericin (白僵菌素)	BEA	[M+NH_4_]^+^	2.98	801.2	134/244.1^*^	/262.2	60/30/30	44
Enniatin B1 (恩镰孢菌素B1)	ENNB1	[M+NH_4_]^+^	2.99	671.3	86.0/196.2^*^	/210.2	60/30/30	40
Enniatin A1 (恩镰孢菌素A1)	ENNA1	[M+NH_4_]^+^	3.25	685.3	100/210.2^*^	/228.2	60/30/30	44
Enniatin A (恩镰孢菌素A)	ENNA	[M+NH_4_]^+^	3.51	699.3	100/210.2^*^	/228.2	60/32/32	42
^13^C_45_-Beauvericin (^13^C_45_-白僵菌素)	^13^C_45_-BEA	[M+NH_4_]^+^	2.98	846.4	259.1		30	44

* Quantitative ion.

## 2 结果与讨论

### 2.1 质谱条件的优化

采用色谱柱进样的方式将质量浓度为500 μg/L的5种标准品分别注入质谱中,一级质谱全扫描,正离子模式下,扫描目标物的特征离子,选择响应值最高的离子作为母离子,分别往流动相中添加酸与铵盐,发现5种待测物均产生[M+H]^+^与[M+NH_4_]^+^离子峰,比较了[M+H]^+^与[M+NH_4_]^+^离子峰响应值的强度,表明[M+NH_4_]^+^离子峰响应值明显高于[M+H]^+^,其中ENNA的[M+NH_4_]^+^离子峰响应强度是[M+H]^+^离子峰的125倍,所以选择[M+NH_4_]^+^离子作为各待测物的母离子(见表1)。按照欧盟2002/657/EC指令,低分辨率质谱检测待测物时应选择两个适宜的子离子的要求,对母离子进行子离子扫描,选择响应值高且信号稳定的子离子为定量离子,响应值稍低的子离子为定性离子,通过仪器自动优化功能优化质谱参数,各待测物子离子的碰撞能量、锥孔电压见表1。

### 2.2 流动相的选择

根据2.1节优化的质谱参数,本实验选取的5种待测物的母离子均为[M+NH_4_]^+^离子峰,在水相中加入铵盐,可以提供更多的NH_4_^+^离子,促进[M+NH_4_]^+^峰的形成,有利于增强母离子强度。分别选取甲醇-甲酸铵溶液、甲醇-乙酸铵溶液、乙腈-甲酸铵溶液和乙腈-乙酸铵溶液4种流动相,考察不同流动相对待测物的色谱分离度、峰形及质谱灵敏度的影响,结果表明,乙腈为有机相时,5种待测物的响应值、灵敏度和峰形明显优于甲醇。其次,比较了水相中添加甲酸铵和乙酸铵对5种待测物的响应值的影响,发现以甲酸铵溶液为水相时,除ENNA的响应值较低外,BEA、ENNA1、ENNB与ENNB1的响应值明显优于乙酸铵溶液,且灵敏度较高。综合考虑,选择乙腈-甲酸铵溶液为流动相,同时优化了甲酸铵溶液的浓度(1、2、5 mmol/L),当甲酸铵浓度为5 mmol/L时,5种待测物的峰形较好,响应值高,梯度洗脱条件下出峰不受杂质干扰,最终确定乙腈-5 mmol/L甲酸铵溶液为流动相。优化后的5种待测物的MRM谱图见图1。

**图1:**
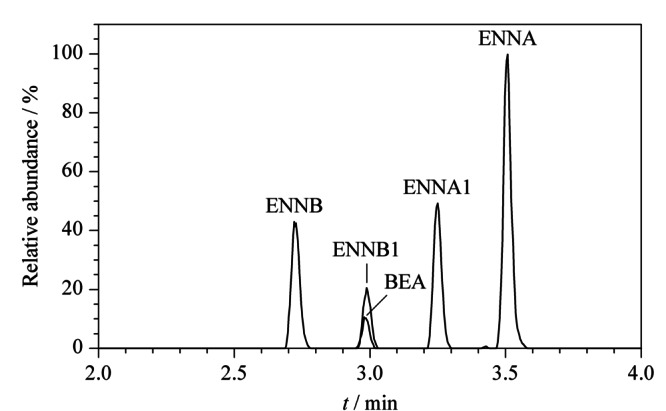
BEA和4种ENNs标准品的总离子流色谱图

### 2.3 样品前处理条件的优化

2.3.1 提取溶剂的选择

提取动物源性食品中的待测物时,甲醇和乙腈是两种常用的有机溶剂,但鸡蛋为高蛋白质样品,纯甲醇与乙腈会使蛋白质变性,样品脱水,迅速结块,影响提取效率。故选取了甲醇-水、乙腈-水、甲醇-水-酸与乙腈-水-酸等溶剂作为提取溶剂。以阴性的鸡蛋作为基质样品,加入5 μg/L的混合标准溶液,通过计算加标回收率考察不同提取溶剂对5种待测物的提取效果,实验发现,采用甲醇-水和甲醇-水-酸为提取溶剂时,样品沉淀物松散,-40 ℃冷冻萃取30 min后,溶解在甲醇中的脂类杂质难析出,甲醇与水相难分层,严重影响了待测物的提取效率,4种ENNs的回收率均比较低,为37.0%~68.6%。采用乙腈-水和乙腈-水-酸为提取溶剂时,5种待测物的回收率明显高于甲醇-水和甲醇-水-酸溶剂(见图2)。文献^[23]^在提取溶剂中加入甲酸,可以提高真菌毒素的稳定性,增加提取效率。本实验比较了乙腈-水溶液中加入1%(v/v)甲酸与1%(v/v)乙酸的提取效果,5种待测物的回收率范围分别为99.2%~151.0%与103%~144%, 1%甲酸与1%乙酸对回收率没有明显差异。但乙腈与水的比例不同会影响回收率大小。

**图2:**
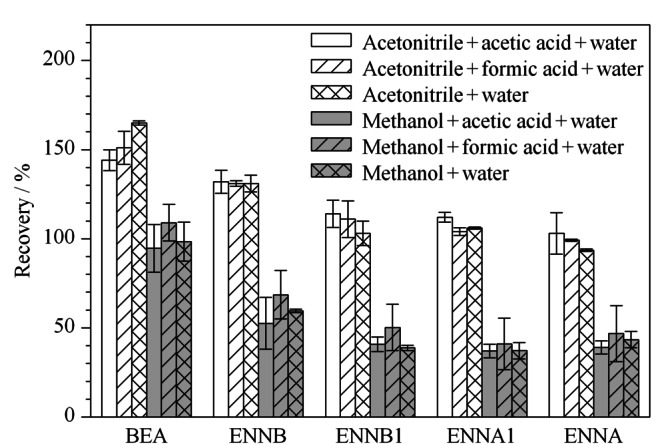
不同提取溶剂对5种待测物平均回收率的影响(*n*=3)

因此,优化了乙腈与水的比例,如图3所示,当乙腈含量为49%~59%时,5种待测物的回收率均大于120%,乙腈含量为69%时,BEA的回收率大于120%, 4种恩镰孢菌素的回收率范围为87%~116%;当乙腈含量为79%, 5种待测物的回收率为73.1%~100.0%;随着乙腈含量增加,5种待测物回收率降低。当乙腈-水溶液中乙腈的比例低时,有机相与水相未达到最佳稳定平衡条件,溶解在乙腈中的脂类等杂质沉淀不完全,产生较强的基质效应。本实验选取的5种待测物极性较小,需要高比例的有机相才能完成溶解,综合考虑,确定乙腈-水-乙酸溶液(79∶20∶1, v/v/v)为最佳提取溶剂,消除了乙腈沉淀蛋白质时致样品结块的现象,涡旋能起到匀浆的效果,同时,避免提取更多脂肪、色素等杂质,使得提取效率更好。

**图3:**
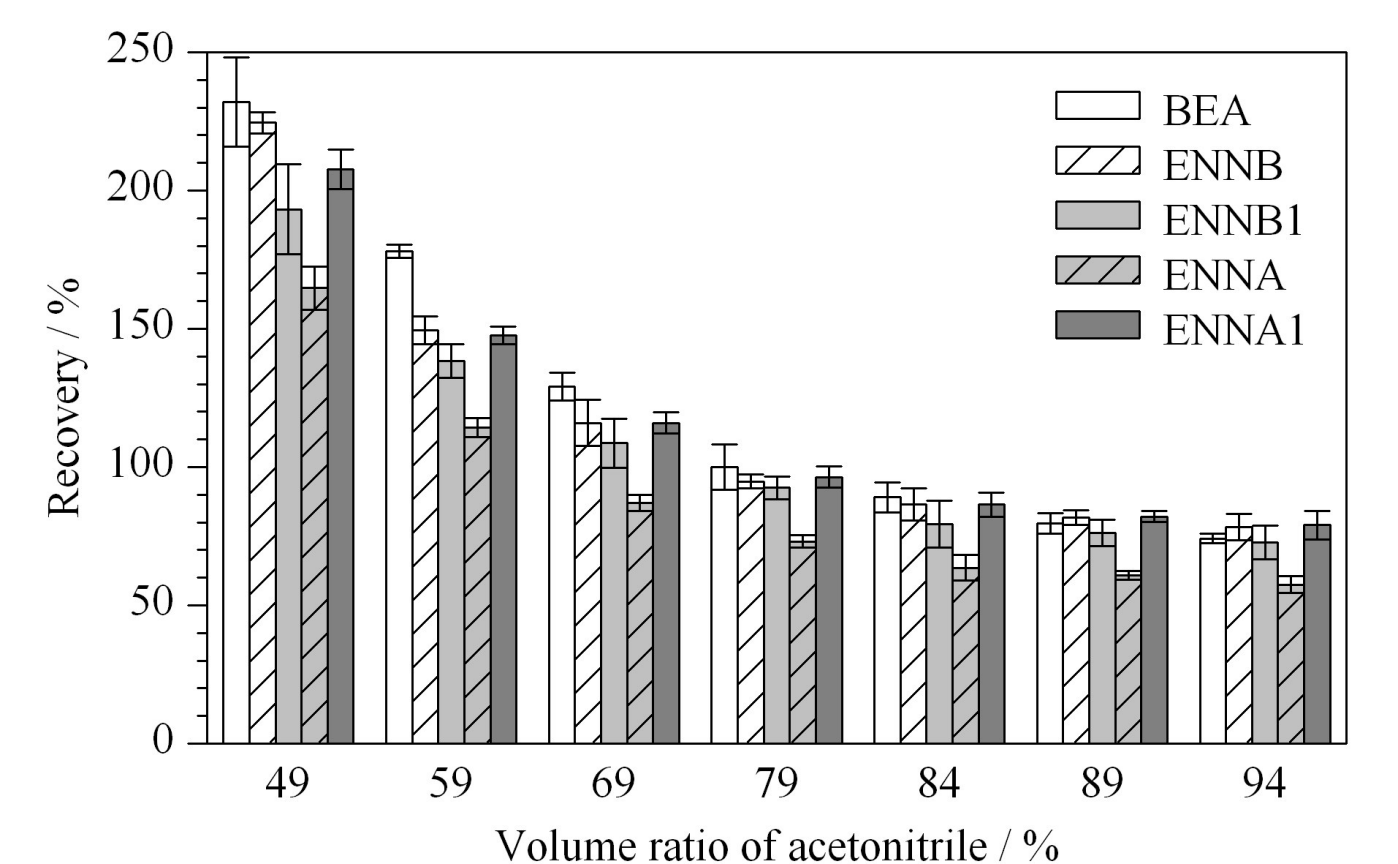
提取溶剂中乙腈的体积分数对5种待测物回收率的影响(*n*=3)

2.3.2 萃取条件的选择

因样品提取液中含有一定量的水会影响后续步骤的浓缩效率,通常考虑在样品提取液中加入一定量的无机盐吸附水或使水与乙腈分层后再取乙腈层浓缩。本实验采用CI-LLE法分离样品提取液中的水与乙腈,需对该法的冷冻温度和时间进行优化,以提高方法的萃取效率,结果表明,采用-20 ℃冷冻时,需要2 h水与乙腈才能明显分层,-30 ℃冷冻时,需要1 h, -40 ℃时,30 min后乙腈与水分层明显,可明显观察到底层溶液有沉淀物析出,且5种待测物的平均加标回收率为90%~101%。乙腈的冰点为-45.7 ℃,若温度选择-45 ℃以下,提取液结冰,影响待测物分配到乙腈层,提取效率低,故选择-40 ℃冷冻30 min作为萃取条件,缩短样品处理时间,提高了浓缩时的效率。

2.3.3 净化剂及含量的选择

提取液-40 ℃下冷冻30 min, 4 ℃ 10000 r/min离心后,取上层2 mL提取液直接浓缩近干、复容后,进样液的颜色较深,无法除去,易对仪器与色谱柱造成污染,5种待测物的平均回收率为128%,超出了70%~120%的范围,说明存在基质效应。因此,离心后的2 mL上层溶液需经DSPE进一步净化。本实验比较了PSA、C18及两者混合使用的净化效果,添加2.5 μg/L的混合标准溶液到阴性鸡蛋样品中,按照1.3节前处理方法提取样品,取2 mL上层溶液,分别加入50mg PSA、50 mg C18和50 mg PSA+50 mg C18等净化剂。结果表明,经C18净化后,5种待测物的回收率为67.0%~87.5%;采用PSA净化时,5种待测物的回收率为69.3%~83.1%,可见,C18与PSA作为净化剂时,5种待测物均能获得较高的回收率,没有明显差异。但经C18净化后的样品液的颜色明显比PSA净化的浅;采用C18与PSA混合净化时,BEA的回收率为86.8%, 4种ENNs的回收率为50.1%~61.2%,回收率较低,两种净化剂同时使用时,对待测物吸附作用较强,影响回收率结果,需要对两者用量比例进行优化。为节省净化剂,本实验选择C18作为净化剂,同时,考察了不同用量的C18对5种待测物加标回收率的影响,当使用70 mg C18作为净化剂时,5种待测物能获得最高的回收率,范围为81.1%~101%,且均在70%~120%范围之内,如图4所示,当净化剂用量大于70 mg时,C18对ENNB1、ENNA和ENNA1有吸附作用,回收率降低,因此,选择70 mg C18作为本实验的净化剂。

**图 4:**
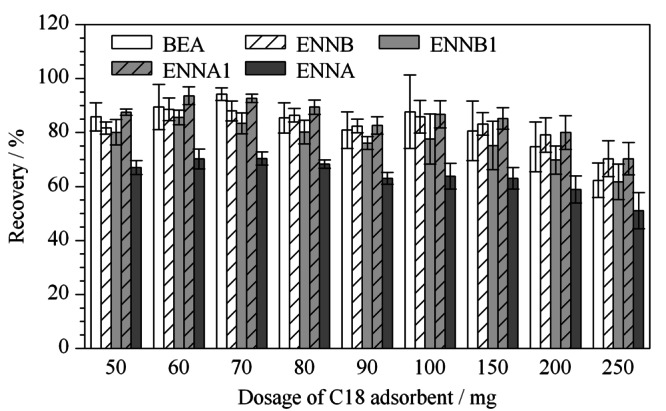
C18净化剂用量对待测物回收率的影响(*n*=3)

### 2.4 基质效应

基质效应(ME)主要是由于样品在离子化时基质成分与目标化合物相互竞争电离而导致目标化合物的质谱信号强度有不同程度的增强或减弱^[31]^,是影响结果准确性的一个重要因素,常被用于评价样品前处理方法,基质效应越强,方法的准确性越低^[32]^。本实验采用1.3节样品前处理方法制备空白鸡蛋基质提取液,然后分别以乙腈-水溶液(80∶20, v/v)和空白鸡蛋基质提取液为溶剂,配制溶剂标准曲线和基质校准曲线,通过公式ME=(基质标准曲线斜率/溶剂标准曲线斜率-1)×100%^[33]^来计算5种待测物的基质效应,以评价基质中干扰物对分析物的影响。结果表明,BEA、ENNB、ENNB1、ENNA1与ENNA的基质效应分别为2.70%、45.1%、24.2%、31.5%与5.16%, 5种待测物的ME均大于0,属于基质增强效应;且ENNB、ENNB1、ENNA1的ME在20%~50%之间,为中等基质效应;因此,本实验在所有样品和标准曲线中加入全碳标记的稳定同位素内标(^13^C_45_-BEA),以内标法定量分析BEA,消除基质效应;对于无法获得稳定同位素内标的4种恩镰孢菌素采用基质匹配标准曲线来消除样品的基质效应,从而保证检测结果的准确性。

### 2.5 方法学验证

2.5.1 线性关系、检出限和定量限

以阴性鸡蛋为基质样品,称取8份,按1.3节方法提取和净化,浓缩后复溶获得空白鸡蛋基质提取液,将8份空白基质提取液合并为一份,混匀后,使用该提取液配制0.1、0.5、1.0、2.0、5.0、10.0、20.0与50.0 μg/L的系列混合标准溶液,加入白僵菌素的同位素内标,使质量浓度为2.5 μg/L,供UPLC-MS/MS分析。待测物BEA采用内标法定量,以待测物与相对应同位素内标的峰面积之比为纵坐标(*y*_IS_),相应的质量浓度为横坐标(*x*, μg/L);待测物ENNA、ENNA1、ENNB与ENNB1采用外标法定量,以待测物的峰面积为纵坐标(*y*),相应的质量浓度为横坐标(*x*, μg/L),绘制基质标准曲线。结果表明,5种待测物在0.1~50.0 μg/L范围内线性关系良好,相关系数(*r*^2^)均大于0.998(见表2)。

**表2:** 5种待测物的线性范围、线性方程、相关系数、检出限及定量限

Analyte	Linear range/(μg/L)	Linear equation	r^2^	LOD/(μg/kg)	LOQ/(μg/kg)
ENNB	0.1-50	y=42028.1x+711.5	0.9997	0.15	0.50
BEA	0.1-50	y_IS_=0.8696x+0.2805	0.9997	0.15	0.50
ENNB1	0.1-50	y=19325.4x+719.1	0.9994	0.10	0.30
ENNA1	0.1-50	y=42921.6x+1580.5	0.9983	0.10	0.30
ENNA	0.1-50	y=88836.4x+3758.6	0.9996	0.05	0.20

y_IS_: peak area ratio of BEA to internal standard; y: peak area; x: mass concentration, μg/L.

在阴性鸡蛋样品中添加不同浓度的混合标准溶液,按照上述前处理方法与色谱条件进行分析,分别以3倍和10倍信噪比(*S/N*)对应的加标浓度定义检出限(LOD)与定量限(LOQ),以LOD与LOQ评估方法的灵敏度,如表2所示,5种待测物的LOD范围为0.05~0.15 μg/kg, LOQ的范围为0.20~0.50 μg/kg。

2.5.2 准确度及精密度

本实验取阴性空白鸡蛋样品,分别添加低、中、高3个浓度水平(0.5、5.0和25.0 μg/kg)的混合标准溶液,按样品前处理方法进行提取与净化,每个加标浓度进行6次重复实验,回收率结果见表3, 5种待测物的平均加标回收率为81.1%~106%,相对标准偏差(RSD)为0.27%~9.79%。

**表3:** 鸡蛋样品中5种待测物的加标回收率和相对标准偏差(n=6)

Analyte	Spiked levels/(μg/kg)				Recoveries/%				RSDs/%		
	Low	Middle	High		Low	Middle	High		Low	Middle	High
ENNB	0.500	5.00	25.0		88.8	96.0	106		1.96	0.27	9.67
BEA	0.500	5.00	25.0		101	97.8	100		3.41	3.48	2.16
ENNB1	0.500	5.00	25.0		81.1	90.6	102		4.45	1.12	5.86
ENNA1	0.500	5.00	25.0		86.8	90.4	99.2		5.61	1.59	9.22
ENNA	0.500	5.00	25.0		87.3	89.0	104		2.80	1.32	9.79

### 2.6 实际样品的测定

应用本方法对随机购买的114份鸡蛋样品(69份来自农村散养户,45份来自市售超市与农贸市场)进行检测,其中4种恩镰孢菌素均未检出,BEA有检出,BEA在农村散养鸡蛋中检出率为30.4%(21/69份),含量范围为0.31~9.82 μg/kg,平均含量1.68 μg/kg, 45份市售鸡蛋中仅1份样品中检出BEA,含量为4.30 μg/kg。鸡蛋阳性样品的TIC色谱如图5所示,待测物BEA检出,4种ENNs的含量低于方法检出限。

**图5:**
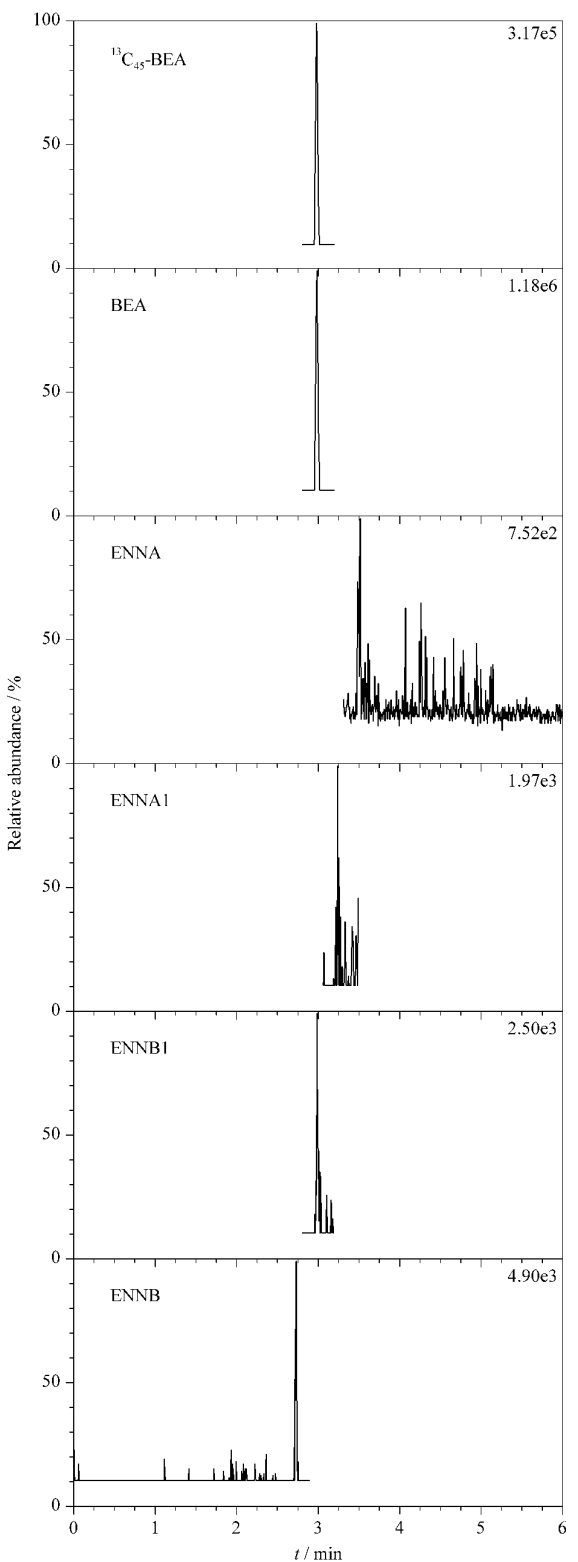
实际鸡蛋样品中BEA的色谱图(9.82 μg/kg)

## 3 结论

本文建立了冷诱导液液萃取-分散固相萃取净化-超高效液相色谱-串联质谱法同时测定鸡蛋样品中白僵菌素与4种恩镰孢菌素的分析方法。对前处理方法与色谱条件进行了优化,并进行了一系列的方法学验证。该方法前处理简单快速,降低了分析成本,提高了检测效率,避免复杂的净化过程中目标物的损失,适合批量禽蛋样品中白僵菌素与恩镰孢菌素快速、准确定量检测。
